# Melatonin Mitigates Kainic Acid-Induced Neuronal Tau Hyperphosphorylation and Memory Deficits through Alleviating ER Stress

**DOI:** 10.3389/fnmol.2018.00005

**Published:** 2018-01-24

**Authors:** Cai Shi, Jia Zeng, Zixi Li, Qingjie Chen, Weijian Hang, Liangtao Xia, Yue Wu, Juan Chen, Anbing Shi

**Affiliations:** ^1^Department of Biochemistry and Molecular Biology, School of Basic Medicine and the Collaborative Innovation Center for Brain Science, Tongji Medical College, Huazhong University of Science and Technology, Wuhan, China; ^2^Department of Clinical Laboratory, Tongji Medical College, Huazhong University of Science and Technology, Wuhan, China; ^3^Institute for Brain Research, Huazhong University of Science and Technology, Wuhan, China; ^4^Key Laboratory of Neurological Disease of National Education Ministry, Tongji Medical College, Huazhong University of Science and Technology, Wuhan, China

**Keywords:** melatonin, kainic acid, tau phosphorylation, endoplasmic reticulum stress, calpain

## Abstract

Kainic acid (KA) exposure causes neuronal degeneration featured by Alzheimer-like tau hyperphosphorylation and memory deficits. Melatonin (Mel) is known to protect hippocampal neurons against KA-induced damage. However, the underlying mechanisms remain elusive. In the current study, we investigated the protective effect of melatonin on KA-induced tau hyperphosphorylation by focusing on endoplasmic reticulum (ER) stress-mediated signaling pathways. By using primary hippocampal neurons and mouse brain, we showed that KA treatment specifically induced ER stress and activated GSK-3β and CDK5, two major kinases responsible for tau phosphorylation. Inhibition of ER stress efficiently inactivated GSK-3β and CDK5. Mechanistically, we found that KA-induced ER stress significantly activated calpain, a calcium-dependent protease. Inhibition of ER stress or calpain leads to the reduction in KA-induced GSK-3β and CDK5 activities and tau phosphorylation. Moreover, GSK-3β or CDK5 inhibition failed to downregulate ER stress efficiently, suggesting that ER stress functions upstream of GSK-3β or CDK5. Notably, our results revealed that melatonin acts against KA-induced neuronal degeneration and tau hyperphosphorylation via easing ER stress, further highlighting the protective role of melatonin in the KA-induced neuronal defects.

## Introduction

Microtubule-associated protein tau functions to promote microtubule assembly and maintains the stability of microtubules (Grundke-Iqbal et al., 1986). Hyperphosphorylated tau protein loses its biological activities and leads to the disruption of microtubules, and thus contributes to the neurodegeneration such as Alzheimer’s disease (AD) and other tauopathies (Hernandez and Avila, 2008). Neurofibrillary tangles (NFTs) is the primary pathological feature of the AD (Morris et al., 2011). The abnormally hyperphosphorylated tau is the primary component of NFTs, which is positively correlated with the decline of memory and cognition in AD patients (Shipton et al., 2011). Therefore, understanding the regulatory mechanisms underlying tau phosphorylation is critical for designing the strategies to arrest AD. Excitatory amino acids have been reported to play a significant role in the pathogenesis of AD (Choi and Rothman, 1990). Kainic acid (KA), an analog of glutamate, has been used to establish excitotoxicity models. Treatment with KA could induce tau hyperphosphorylation and neuronal death through activating GSK-3β and CDK5 pathway in both *in vivo* and *in vitro* models (Liang et al., 2009; Tripathi et al., 2010). GSK-3β and CDK5 are two major tau kinases involved in AD-like tau hyperphosphorylation, while the precise intracellular mechanisms through which KA induces tau hyperphosphorylation via GSK-3β and CDK5 activation are still poorly understood.

Endoplasmic reticulum (ER) stress constitutes early events in AD pathogenesis and has been associated with AD progression (Salminen et al., 2009). In the temporal cortex and hippocampus of AD patients, increased level of GRP78 was observed (Hoozemans et al., 2005). The PERK was also found to be abundant in neurons of AD patients (Hoozemans et al., 2009). The over-activation of glutamate receptors by KA treatment compromises the ER homeostasis and eventually leads to activation of ER stress (Zhang and Zhu, 2011). In addition, ER stress inhibitor salubrinal can attenuate KA-induced hippocampal cell death (Kim et al., 2014), suggesting that KA is capable of inducing ER stress.

Melatonin (Mel), a tryptophan metabolite, is synthesized by the pineal gland and many other organs (Acuna-Castroviejo et al., 2014). Melatonin is well known for its roles in the regulation of circadian rhythms, sleep, mood, reproduction, tumor growth, and aging (Wu and Swaab, 2005). Deficiency of melatonin in the cerebrospinal fluid (CSF) and blood is highly correlated with the progression of cognitive impairment (Liu et al., 1999). For patients suffering from the very early stage of the AD, the decline of melatonin in CSF and blood serve as a sensitive indicator of cognitive dysfunction (Zhou et al., 2003). Importantly, melatonin supplementation can effectively slow down the progress of hippocampus neuronal degeneration and improve the cognition ability (Brusco et al., 2000). Furthermore, melatonin was reported to regulate the activities of CDK5 and GSK-3β in hippocampal neurons (Peng et al., 2013). Accumulating evidence suggests that the neuroprotective effect of melatonin is associated with the modulation of ER stress. For instance, melatonin was used to alleviate ER stress in a rat model of renal warm ischemia reperfusion (Hadj Ayed Tka et al., 2015). In murine splenocytes, melatonin reversed ER stress-mediated apoptosis induced by the herbicide atrazine (Sharma et al., 2014). Constant light illumination increased expression of GRP78 in rats, supplement with melatonin could efficiently protect the cells from the ER stress-induced impairments (Ling et al., 2009). Consistently, in the current study, we show that KA affects CDK5/GSK-3β and tau phosphorylation via ER stress induction. Melatonin executes protective effects against KA-induced neuronal excitotoxicity through ER stress alleviation.

## Materials and Methods

### Cell Culture and Treatments

Primary hippocampal neurons were prepared from 2-day-old Sprague-Dawley rats using the described protocols, with modifications (Isaev et al., 2002). The tissue was briefly digested with 0.25% trypsin in phosphate-buffered saline (PBS) for 20 min at 37°C followed by mechanical dissociation. Primary hippocampal neurons were seeded in poly-L-lysine-coated plates (120,000 cells/cm^2^) and grown in neurobasal medium with B-27 serum-free supplement (Gibco, Grand Island, NY, United States), 100 U/mL penicillin, 100 g/mL streptomycin, and 2 mM L-glutamine. The cultures were maintained in a humid incubator aerated with 95% air and 5% CO_2_ at 37°C. The medium was changed starting from the 4th day by replacing half of the medium twice a week. Serum-free primary neuron cultures were utilized for the experiments after 8 days.

In experiment 1, the primary hippocampal neurons were pre-treated with or without melatonin (50 μM) for 1 h and then stimulated with KA (50 μM) or thapsigargin (TG, 2 μM, Sigma, St. Louis, MO, United States) for 8 h. Melatonin (Sigma, St. Louis, MO, United States) was first dissolved in absolute ethanol at a concentration of 50 mM and diluted with culture medium to the final concentration. KA (Abcam, Cambridge, MA, United States) was first dissolved in dimethyl sulfoxide (DMSO) at a concentration of 100 mM and then diluted with culture medium to the final concentration. TG was dissolved in DMSO at a concentration of 1 mM as stock solutions and then diluted with medium to 2 μM. Corresponding dilutions of ethanol and DMSO were given to the control group.

In experiment 2, the primary hippocampal neurons were pre-treated with or without Salubrinal (Sal, an ER stress inhibitor, 5 or 10 μM, Sigma, St. Louis, MO, United States), SB216763 (SB, a specific GSK-3β inhibitor, 2 or 20 μM, Tocris Bioscience, Bristol, United Kingdom), roscovitine (Ros, a CDK5 inhibitor, 50 or 100 μM, Sigma, St. Louis, MO, United States) and MDL28170 (MDL, a calpain inhibitor, 10 μM, Sigma, St. Louis, MO, United States) for 1 h, and then stimulated with KA (50 μM) for 8 h. Sal, SB, and Ros were dissolved in DMSO as stock solutions at the concentration of 20 mM, then further diluted into cell culture medium to a final concentration as described before. A stock solution of the MDL was prepared in DMSO at the concentration of 5 mM then further diluted into cell culture medium to the final concentration of 10 μM. Corresponding dilutions of DMSO were given to control, and KA-treated group.

### Animals and Treatments

Adult male C57BL/6 mice, weighing 25 ± 2 g, were supplied by the Experimental Animal Center of Tongji Medical College. All experimental procedures were approved by the Animal Care and Use Committee at the Huazhong University of Science and Technology and were performed in compliance with National Institutes of Health guidelines on the ethical use of animals. The mice were housed five per cage in a room maintained at 22 ± 2°C with an alternating 12-h light-dark cycle. Food and water were available *ad libitum*.

In experiment 1, based on the previous study, a total of 42 mice were treated with an intraperitoneal (i.p.) injection of 30 mg/kg KA (Abcam, Cambridge, MA, United States) emulsified in 0.9% normal saline (Crespo-Biel et al., 2010). To validate the neurotoxic induction of KA, behavioral observations were made every 30 min for 4 h after KA injection. The mice which did not display general limbic seizure activity within 90 min after the KA injection were excluded from further study. Thus, the final numbers of KA groups were 35. Mice in the control group (*n* = 7) were injected with saline. Animals in the experimental group were sacrificed 6 and 12 h and 1, 3, and 7 days after KA treatment.

In experiment 2, a total of 36 mice were divided randomly into three groups: KA-only group (KA), melatonin (Sigma, St. Louis, MO, United States) administration before KA group (Mel+KA), and vehicle-treated control group (Con). Based on the previous study (Jain et al., 2013), melatonin was dissolved in absolute ethanol and diluted in saline to a final concentration before injection. The mice in the Mel+KA group were given i.p. injections of 20 mg/kg melatonin once 30 min before the injection of KA on the 1st day and a single dose per day for a total of 3 days (Jain et al., 2013). The mice in each group were sacrificed on the 4th day after KA treatment. All mice were sacrificed after KA treatment, and the hippocampal tissue was harvested for further tests.

### Morris Water Maze Test

To assess the cognitive changes, Morris water maze (MWM) test was conducted in a circular water tank (120 cm in diameter and 35 cm in height) filled with water to the depth of 21 cm and maintained at 25 ± 1°C. The tank was divided into four equal quadrants. A submerged square platform (15 cm in width and 20 cm in height) was placed in the third quadrant of the tank with its top surface 1 cm below the water surface. The mice were placed in the pool at four possible start locations facing the wall of the pool, and a camera was simultaneously activated. Each mouse was allowed up to 60 s to locate the platform. The trial was terminated when the mouse found the platform within 60 s. If a mouse failed to find the platform within 60 s, it was guided by a researcher to locate the platform and allowed to stay for 2–3 s. Each mouse was conditioned three times per day for 2 days to let them adapt to the pool environment (visible platform training) and then tested three times per day for 6 days to find the hidden platform (hidden platform training). The latency (the time taken to locate the platform in water), distance, and swim speed were recorded using an automated video tracking software package (NoldusEtho Vision 2.3.19, Netherlands). For data analysis, the pool was divided into four quadrants. During the visible platform training, the platform was moved to a different quadrant for each session. During the hidden platform training, the platform location was identical to each mouse. Behaviors of the mice were tracked using EthoVision 3.0, and the escape latency was analyzed. In each trial, the average latency time for each mouse was calculated and recorded.

### Western Blotting

Total proteins were extracted by using a protein extraction kit (Pierce, IL, United States) following the manufacturer’s protocol. Protein extracts were dissolved in 10–15% sodium dodecyl sulfate polyacrylamide gel and then transferred to a nitrocellulose membrane at 150 mA. After being blocked with 5% non-fat skimmed milk [diluted with Tris-buffered saline containing 0.1% Tween 20 (TBST)] for 1 h at room temperature, the membrane containing the protein extracts was incubated overnight with primary antibody (diluted with 2% bovine serum albumin in TBST) at 4°C. The following primary antibodies were used: anti-β-actin (1:3000, Santa Cruz, CA, United States), anti-tau/ps396 (1:1000, Abcam, Cambridge, MA, United States), anti-tau/ps199 (1:1000, Abcam, Cambridge, MA, United States), and anti-tau-5 (1:1000, Abcam, Cambridge, MA, United States); anti-CDK5 (1:1000, Santa Cruz, CA, United States); anti-GSK-3β (1:1000, CST, Framingham, MA, United States); anti-calpain (1:1000, Santa Cruz, CA, United States); and anti-p35 (1:1000, Santa Cruz, CA, United States), anti-GRP78 (1:2000, Abcam, Cambridge, MA, United States); anti-ATF-6 (1:1500, Abcam, Cambridge, MA, United States); anti-ATF-4 (1:1000, Abcam, Cambridge, MA, United States); anti-IRE-1 (1:1500, Abcam, Cambridge, MA, United States); anti-eIF-2α (1:1000, CST, Framingham, MA, United States) and anti-p-PERK (1:500, Santa Cruz, CA, United States). On the 2nd day, proteins were visualized using the enhanced chemiluminescence detection system (Pierce, IL, United States) after incubating with corresponding secondary antibodies (1:1000, Amersham Pharmacia Biotech, Buckinghamshire, United Kingdom) and then exposed to medical x-ray film. For the quantitative analysis of band intensity, we calculated the band intensity ratio for normalization by using a gel image analyzer (JS380; Peiqing Science and Technology, Shanghai, China). For each blot, multiply the background subtracted density of the target protein in each lane by the ratio of the density of the loading control (such as housekeeping protein) from a control sample of all the study blots to the other lanes in the gel. This will give the normalized density to the loading control (NDL). Calculate the fold difference for each replicate by dividing NDL from each lane by the NDL from the control sample.

### Immunochemistry

Mouse hippocampal tissues were frozen and sectioned consecutively at 10 μm, with three sections for each embedded sample. The sections were incubated overnight at 4°C with tau ps-396 antibody (1:1000), respectively. After incubation with horseradish peroxidase-conjugated secondary antibody (Jackson Immuno Research, West Grove, PA, United States) for 2 h at room temperature, sections were washed and developed with 0.05% diaminobenzidine (DAB) plus 0.015% hydrogen peroxide in PBS. The DAB-stained sections were air-dried, counterstained with Mayer’s hematoxylin, dehydrated, cleared, and coverslipped. Finally, the sections were visualized with DAB for light microscopy examination. For every section, DG, CA1, and CA3 areas of the hippocampus were examined with an Olympus AX-70 microscope equipped with a motorized stage (Olympus, NY, United States). Images were captured at 20× magnification. Image analysis of all sections was performed within 4 weeks after immunohistochemistry with the tau ps-396 antibody. For quantification, we used the Fiji/ImageJ open source software to perform digital slide image processing. To overcome potential heterogeneity in intensities across different in-slide areas, we calculated the mean reciprocal intensity (RI) of a region of interest (ROI). A uniformly sized ROI was placed over the mossy fibers staining using the ‘Draw’ tool, and a standard RBG (red–green–blue) image was produced from each respective slide, by applying DAB-specific color deconvolution. Mean intensity was calculated using the ‘Measure’ function under the ‘Analyze’ menu of the Fiji software. Since the maximum intensity value of an RGB image analyzed in Fiji is 250, we subtracted the mean intensity of each stained ROI from 250, thereby deriving a RI that was directly proportional to the amount of chromogen present.

### Enzyme Activity Assay

The calpain activity was measured using Calpain Substrate II Kit (Merck Millipore, Darmstadt, Germany). Cells were washed twice with PBS and lysed on ice with extraction buffer. The total protein extracted (50 μg) was incubated with 150 μL substrate and 1 mL reaction buffer for 100 min at 37°C. The release of 7-amino-4-methyl-coumarin (AMC) from the reaction was monitored at 440 nm of emission using a fluorescence spectrometer. Fluorescence units were converted into AMC release using standard curve. The activity of calpain was expressed and documented in pmol of AMC cleaved per minute per milligram of protein.

GSK-3 activity was assessed using γ-^32^P-ATP assay. The reaction mixture contained 30 mM Tris-HCl (pH 7.4), 10 mM MgCl_2_, 10 mM NaF, 1 mM Na_3_VO_4_, 2 mM EGTA, 0.2 mM ATP, 2000 cpm/pmol γ-^32^P-ATP, 10 mM β-mercaptoethanol, and 7.5 μg protein sample. After incubating for 30 min at 30°C, the reaction was terminated by adding 300 mmol/L H_3_PO_4_. The γ-^32^P-labeled peptide was blotted on a phosphor-cellulose paper, then washed with 75 mmol/L H_3_PO_4_ three times and dried at 60°C overnight. Incorporation of γ-^32^P was quantified in counts per minute (cpm) using a scintillation counter.

CDK-5 activity was assayed using the procedure described by Pigino et al. (1997). A sample containing 200 μg total cellular protein was used for immunoprecipitation with the anti-CDK-5 antibody (1:50) and Protein G Plus-Agarose (Santa Cruz, CA, United States). The immunoprecipitates were rinsed three times with lysis buffer and once with kinase buffer (50 mM HEPES, pH 7.0, 10 mM MgCl_2_, 1 mM DTT, and 1 mM cold ATP). The rinsed agarose beads were incubated with kinase buffer containing 2.5 μg histone H1 plus 200 μMγ-^32^P-ATP in a final volume of 50 μL for 30 min at 30°C, and the reaction products were determined using a scintillation counter.

### Measurement of Malondialdehyde and Superoxide Dismutase

Cells were seeded in 6-well culture plates. After 8 h of treatment with KA or Mel+KA, cells were washed twice with PBS (pH 7.4, 4°C) and lysed with buffer containing 10 mM Tris-HCl (pH 7.4), 10 mM ethylenediaminetetra-acetic acid and 0.2% Triton X-100 on ice. The cells were scraped from the wells and centrifuged at 17000 × *g* for 15 min at 4°C. Superoxide dismutase activity in the supernatant was determined by assessing the inhibition of pyrogallolautoxidation (Marklund and Marklund, 1974).

Levels of Malondialdehyde (MDA), a metabolite of lipid peroxides, were estimated as reactive substances by thiobarbituric acid addition (Kobe et al., 2002). Primary hippocampal neurons were washed twice and collected in cold PBS and centrifuged at 3000 × *g* for 5 min. Then, the pellets were thoroughly mixed with 4 mL of 0.083 M sulfuric acid and 0.5 mL of 10% phosphotungstic acid. After centrifugation at 3000 × *g* for 10 min, the liquid phase was decanted. Four milliliters of double-distilled water and thiobarbituric acid reagent (0.67% 2-thiobarbituric acid/acetic acid 1:1) were added to each sample, mixed, and heated in a water bath at 95°C for 1 h. The samples were cooled with tap water, 5 mL of N-butanol-alcohol was added. The samples were vigorously shaken for 1 min and centrifuged. The N-butanol–alcohol phase, which contains the lipid peroxides, was used for MDA analysis with a fluorospectrophotometer (F-2000, Hitachi Ltd., Tokyo, Japan) using excitation/emission wavelengths of 515/553 nm. Freshly diluted tetramethoxypropane, which yields MDA, was used as a standard, and the results are expressed as nanomoles of MDA equivalents. The protein concentration was measured using the BCA reagent (Pierce, FL, United States).

### Flow Cytometric Measurement of Cytosolic Ca^2+^ Concentration ([Ca^2+^]c) and Endoplasmic Reticulum Ca^2+^ Levels ([Ca^2+^]_ER_)

Intracellular Ca^2+^ concentration was measured using the calcium indicator Fura-2 AM. After treatment, primary hippocampal neurons grown on glass slides were loaded with 5 μM Fura-2 AM for 45 min in Hanks Balanced Salt Solution (HBSS), and equilibrated for 30 min in the dark at room temperature. Cells were then placed in an open-bath imaging chamber containing HBSS. Using a Nikon inverted epifluorescence microscope, neurons were excited at 345 and 385 nm and the emission fluorescence at 510 nm was recorded. Images were collected and analyzed with the MetaFluor image-processing software, and the results were calculated and reported as *F*_t_/*F*_0_ and area under curve (AUC).

It has been verified that Mag-Fluo-4-AM was selectively labeled on ER, making its specificity for measuring [Ca^2+^]_ER_ (Wang et al., 2015). After corresponding treatment, cells were incubated with 2 μM Mag-Fluo-4-AM and 0.02% (w/v) Pluronic F-127 for 30 min at 37°C in dark. 488 nm laser was used to excite Mag-Fluo-4 fluorescence and the emission fluorescence at 510 nm was recorded. Images were collected and analyzed with the MetaFluor image-processing software.

### Statistical Analysis

Data were expressed as the mean ± standard deviation and analyzed using SPSS 10.0 statistical software (SPSS Inc., Chicago, IL, United States). The one-way and two-way ANOVA tests were used to determine the significance of differences among groups (*P* < 0.05, *P* < 0.01, *P* < 0.001).

## Results

### Kainic Acid Treatment Boosts Tau Phosphorylation in Hippocampal Neurons

**FIGURE 1:**
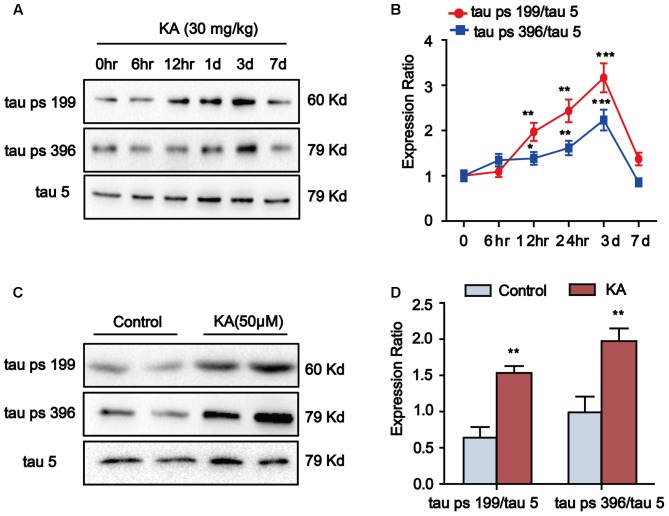
Kainic acid (KA) augments tau phosphorylation level *in vivo* and *in vitro*. **(A,B)** Phosphorylation levels of tau (Ser199 and Ser396) in KA-treated mouse hippocampus at different time points. **(C,D)** Phosphorylation levels of tau (Ser199 and Ser396) in KA-treated primary hippocampal neurons. (^∗∗^*P* < 0.01, ^∗∗∗^*P* < 0.001 vs. controls; the significant difference from the respective values were determined by one-way analysis of variance test. *N* = 3 for western blotting).

Kainic acid (KA) has been known to cause status epileptics, neurodegeneration and memory loss (Milatovic et al., 2001). To validate the neuronal toxicity of KA, we treated the mouse with i.p. injections (30 mg/kg KA; 6 and 12 h and 1, 3, and 7 days) and measured the hippocampal phosphorylation level of tau protein. In the groups of 12-h, 1- and 3-day after KA treatment, we observed the significant increase in tau phosphorylation at Ser199 and Ser396 in the hippocampus. Seven days after KA administration, there were no further changes in tau phosphorylation (**Figures 1A,B**). Thus, we harvested hippocampus tissue 3 days after KA treatment for the experiments.

We further assayed the tau phosphorylation in primary hippocampal neurons. Eight hours after KA treatment, we noticed that the tau phosphorylation at Ser199 and Ser396 sites significantly increased in primary hippocampal neurons and the level of tau phosphorylation reached the highest point at 16-h (**Figures 1C,D**). Considering the efficiency of melatonin treatment (Xue et al., 2017), we chose to use the 8-h time point in our assays. It is worth noting that the phosphorylation level of tau started to increase after 12 h of KA treatment and reached the peak level at 3-day in the animal model (**Figures 1A,B**). We speculate that the tau phosphorylation boost latency is due to the late onset time of KA in the live animals. Taken together, our observations indicated that KA treatment could efficiently boost tau phosphorylation both *in vivo* and *in vitro*.

### Melatonin Mitigates Kainic Acid-Induced Memory Deficits and Tau Hyperphosphorylation

**FIGURE 2:**
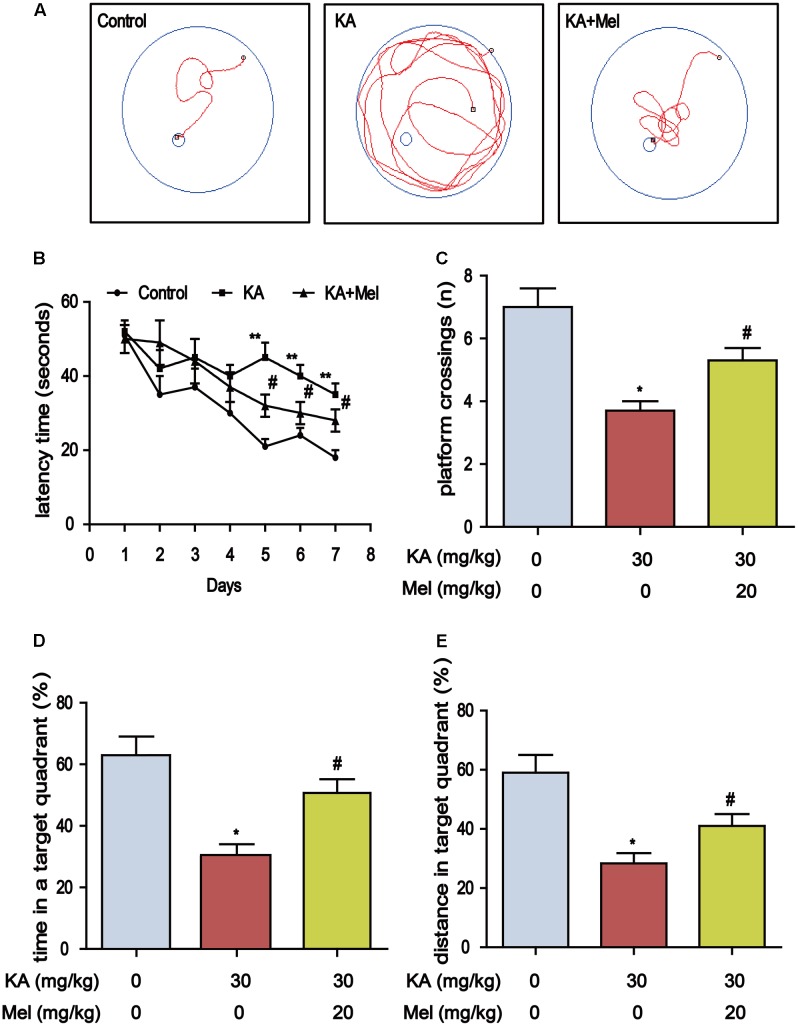
Melatonin mitigates KA-induced memory deficits by using Morris Water Maze test. **(A)** The trajectory of rats in MWM. **(B)** Escape latency analysis in the training trials. **(C)** The frequency of crossing over the former location during the probe trial. **(D)** The percentage of time spent in target quadrant during the probe trial. **(E)** The percentage of distance in the target quadrant during the probe trial. (^∗^*P* < 0.05, ^∗∗^*P* < 0.01 vs. the control group; ^#^*P* < 0.05 vs. the KA-only group, *n* = 7 per group for MWM test; the significant difference from the respective values were determined by two-way analysis of variance test).

Deficiency of melatonin in the CSF and blood is highly correlated with the progression of cognitive impairment (Liu et al., 1999). Melatonin supplementation slows down neuronal degeneration progression in rat hippocampus and improves cognition ability (Zhou et al., 2003). To assess whether melatonin could alleviate KA-induced memory impairment, we performed MWM test to assay the learning ability of the mouse via training with a hidden platform. We observed that the mean escape latency in KA-only group (F6, 84 = 15.16, *P* < 0.001) increased which compared to control group, and the escape latency of Mel+KA group was shorter than that of KA-only group (F6, 84 = 9.214, *P* < 0.001) (**Figures 2A,B**). On the 7th day, we removed the platform and conducted the probe trial. The platform crossing frequency of Mel+KA group was higher than those of KA-only group (**Figure 2C**). The mice from KA-only group spent less time in the target quadrant than those from control group did, while the mice from Mel+KA group spent comparable time with control group animals in the target quadrant (**Figure 2D**). Also, the percent distance of Mel+KA group in the target quadrant increased significantly (**Figure 2E**). Taken together, these results demonstrated that melatonin ameliorates KA-induced memory deficits.

**FIGURE 3:**
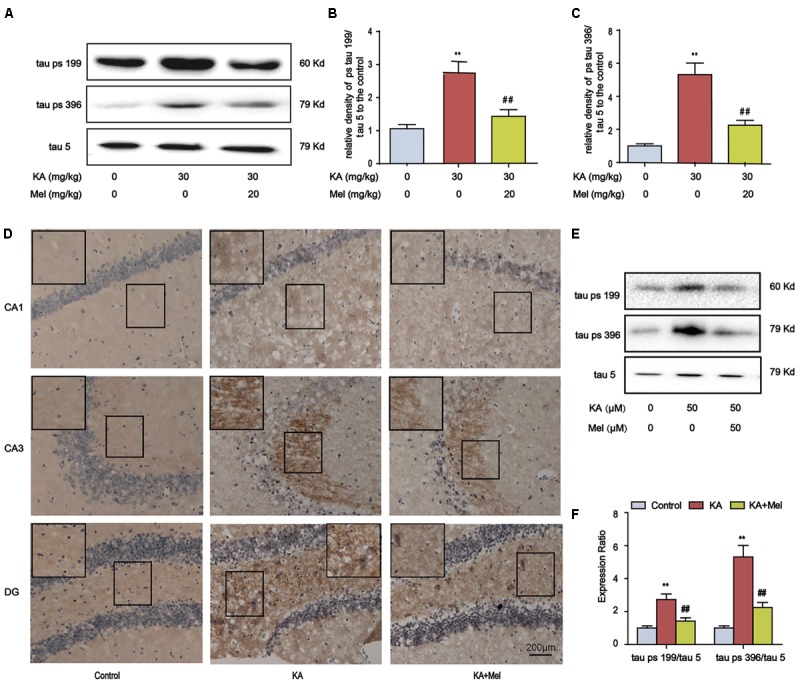
Melatonin ameliorates KA-induced tau hyperphosphorylation. **(A–C)** Phosphorylation levels of tau (Ser199 and Ser396) in KA and/or Mel-treated mouse hippocampus. The KA group mice were given were given i.p. injection of 30 mg/kg KA. The Mel+KA group mice were given i.p. injections of 20 mg/kg melatonin once 30 min before the injection of 30 mg/kg KA on the 1st day and a single dose per day for a total of 3 days. **(D)** Immunohistochemical staining of tau phosphorylation at Ser396 in KA and/or Mel-treated mouse hippocampus. **(E,F)** Phosphorylation levels of tau (Ser199 and Ser396) in KA and/or Mel-treated primary hippocampal neurons. The primary hippocampal neurons were pre-treated with or without melatonin (50 μM) for 1 h and then stimulated with KA (50 μM) for 8 h. (^∗∗^*P* < 0.01 vs. the control group; ^##^*P* < 0.01 vs. the KA-only group, *n* = 7 per group for immunohistochemical staining test; *n* = 3 for western blotting; the significant difference from the respective values were determined by one-way analysis of variance test).

Memory impairment is closely associated with tau hyperphosphorylation (Medeiros et al., 2011), we speculated that tau phosphorylation alleviation might underlie the protective effect of melatonin. Indeed, melatonin treatment significantly lowered the tau phosphorylation at Ser396 and Ser199 in KA-treated mice (**Figures 3A–C**). We further assayed the distribution of Ser396 hyperphosphorylated tau. KA treatment increased the staining density of p-tau Ser396 in CA1, CA3, and DG regions. The stronger immunoreactivity in the mossy fibers in CA3 sector suggested that the neurons in CA3 region are more sensitive to KA, while the KA-sensitivity of CA1 and DG neurons are relatively mild. Alternatively, these data suggested that the signals of KA may be activated differentially in various regions of the hippocampus. Notably, melatonin decreased the staining density in the CA1, CA3, and DG regions of KA-treated mice hippocampus (**Figure 3D**). Also, melatonin significantly reduced the phosphorylation level of tau in KA-treated primary hippocampal neurons (**Figures 3E,F**).

### Kainic Acid-Induced Tau Hyperphosphorylation Is Highly Correlated with ER Stress

**FIGURE 4:**
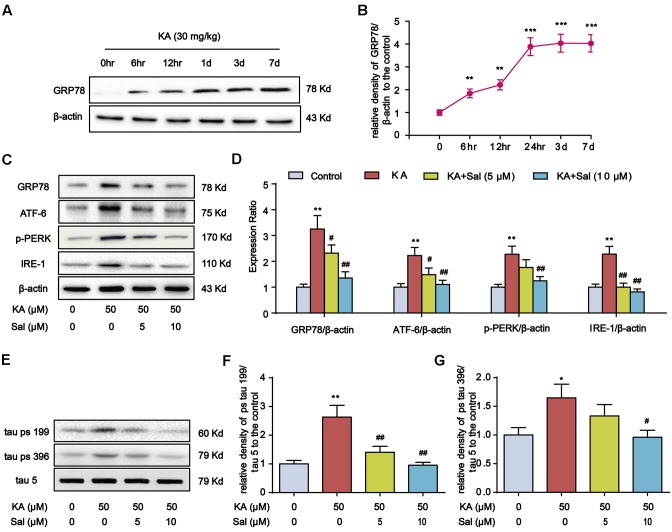
Kainic acid-induced tau hyperphosphorylation is highly correlated with ER stress. **(A,B)** Protein levels of GRP78 in KA-treated mouse hippocampus at different time points. **(C,D)** Protein levels of GRP78, ATF-6, p-PERK, and IRE-1 in KA and/or Sal-treated primary hippocampal neurons. The primary hippocampal neurons were pre-treated with or without Salubrinal (Sal, an ER stress inhibitor, 5 or 10 μM) for 1 h and then stimulated with KA (50 μM) for 8 h. **(E–G)** Phosphorylation levels of tau (Ser199 and Ser396) in KA and/or Sal-treated primary hippocampal neurons. (^∗^*P* < 0.05, ^∗∗^*P* < 0.01, ^∗∗∗^*P* < 0.001 vs. controls; ^#^*P* < 0.05; ^##^*P* < 0.01 vs. the KA group; the significant difference from the respective values were determined by one-way analysis of variance test. *N* = 3 for western blotting).

Previous reports showed that ER chaperone protein GRP78 level increases in AD patient brains (Hoozemans et al., 2005). Consistently, the GRP78 level rose in KA-treated mouse hippocampus and reached the highest level at day 7 (**Figures 4A,B**). Then, we pretreated primary hippocampal neurons with ER stress inhibitor Salubrinal (Sal) for 1 h and then stimulated with KA for 8 h. With the addition of Sal, the expression levels of GRP78, ATF-6, p-PERK, and IRE-1 significantly decreased (**Figures 4C,D**), indicating that KA treatment can effectively induce ER stress.

Our recent study showed that ER stress plays an essential role in KA-induced neuronal death (Xue et al., 2017). Thus, we reasoned that ER stress might contribute to KA-induced tau hyperphosphorylation. In parallel with ER stress biomarkers change, tau phosphorylation at Ser199, and Ser396 sites were down-regulated significantly in KA+Sal neurons (5 and 10 μM) (**Figures 4E–G**), suggesting that KA-induced ER stress modulates tau phosphorylation.

### Kainic Acid-Induced ER Stress Promotes GSK-3 and CDK5 Activities

**FIGURE 5:**
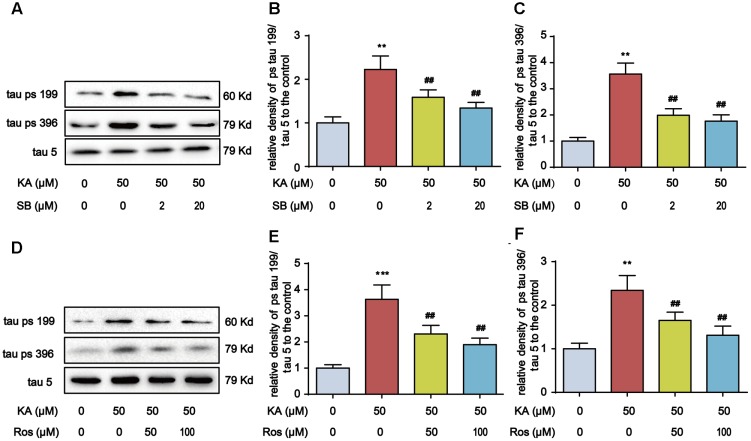
SB216763 (SB)/Roscovitine (Ros) inhibits tau hyperphosphorylation in primary hippocampal neurons. **(A–C)** Phosphorylation levels of tau (Ser199 and Ser396) in KA and/or SB-treated primary hippocampal neurons. The primary hippocampal neurons were pre-treated with or without SB216763 (SB, a specific GSK-3β inhibitor, 2 or 20 μM) for 1 h and then stimulated with KA (50 μM) for 8 h. **(D–F)** Phosphorylation levels of tau (Ser199 and Ser396) in KA and/or Ros-treated primary hippocampal neurons. The primary hippocampal neurons were pre-treated with or without roscovitine (Ros, a CDK5 inhibitor, 50 or 100 μM) for 1 h and then stimulated with KA (50 μM) for 8 h. (^∗∗^*P* < 0.01, ^∗∗∗^*P* < 0.001 vs. controls; ^##^*P* < 0.01 vs. KA group; the significant difference from the respective values were determined by one-way analysis of variance test. *N* = 3 for western blotting).

**FIGURE 6:**
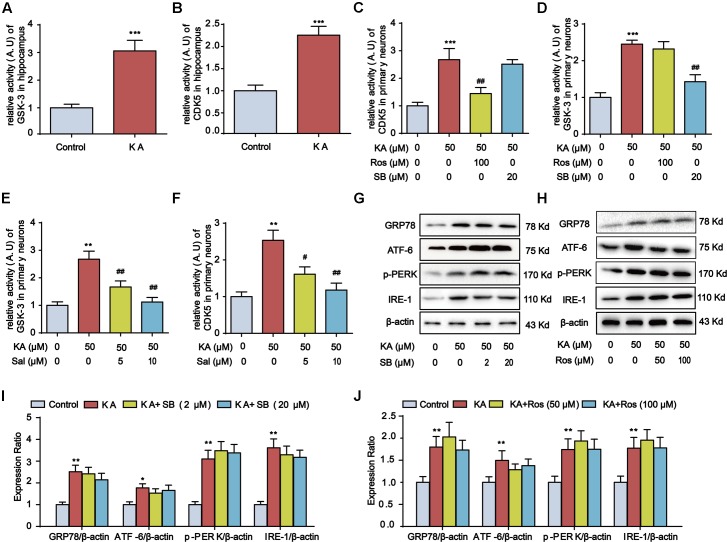
Kainic acid-induced ER stress promotes GSK-3 and CDK5 activities. **(A)** Relative activity of GSK-3 in KA-treated mouse hippocampus. **(B)** Relative activity of CDK5 in KA-treated mouse hippocampus. **(C)** Relative activity of CDK5 in KA, KA+Ros, and/or KA+SB -treated primary hippocampal neurons. The primary hippocampal neurons were pre-treated with or without SB (20 μM) or Ros (100 μM) for 1 h and then stimulated with KA (50 μM) for 8 h. **(D)** Relative activity of GSK-3 in KA, KA+Ros, and/or KA+SB -treated primary hippocampal neurons. **(E)** Relative activity of GSK-3 in KA and/or Sal-treated primary hippocampal neurons. The primary hippocampal neurons were pre-treated with or without Salubrinal (5 or 10 μM) for 1 h and then stimulated with KA (50 μM) for 8 h. **(F)** Relative activity of CDK5 in KA and/or Sal-treated primary hippocampal neurons. **(G,I)** Protein levels of GRP78, ATF-6, p-PERK, and IRE-1 in KA and/or SB-treated primary hippocampal neurons. **(H,J)** Protein levels of GRP78, ATF-6, p-PERK, and IRE-1 in KA and/or Ros-treated primary hippocampal neurons. (^∗^*P* < 0.05, ^∗∗^*P* < 0.01, ^∗∗∗^*P* < 0.001 vs. controls; ^#^*P* < 0.05; ^##^*P* < 0.01 vs. the KA group; the significant difference from the respective values were determined by one-way analysis of variance test. *N* = 3 for western blotting; *n* = 5 for assay of activity).

GSK-3β and CDK5 are two major protein kinases implicated in AD-like tau hyperphosphorylation, and KA-induced tau hyperphosphorylation is directly associated with the increase in GSK-3β and CDK5 activities (Tripathi et al., 2010). To assay the requirement of GSK-3 and CDK5 in KA-induced tau hyperphosphorylation, we deployed SB216763 (SB, a GSK-3 specific inhibitor) and roscovitine (Ros, a CDK5 specific inhibitor) in KA-treated primary hippocampal neurons, and found that both SB and Ros inhibited tau hyperphosphorylation (**Figure 5**). The activity of CDK5 and GSK-3 increased by ∼2- and ∼3-fold 3 days after KA injection, respectively (**Figures 6A,B**). Consistently, the activities of CDK5 and GSK-3 were inhibited by roscovitine or SB216763 in KA-treated primary hippocampal neurons, respectively (**Figures 6C,D**). Notably, in KA and ER stress inhibitor Salubrinal co-treated primary hippocampal neurons, GSK-3 and CDK5 activities were reduced (**Figures 6E,F**), implying the functional role of ER stress in GSK-3 and CDK5 activities regulation. Furthermore, the upregulated ER stress biomarkers after KA treatment remained intact upon the addition of SB or Ros (**Figures 6G–J**), suggesting that ER stress acts upstream of GSK-3 and CDK5 in the process of tau hyperphosphorylation.

### Calpain Mediates Kainic Acid-Induced GSK-3 and CDK5 Activation

**FIGURE 7:**
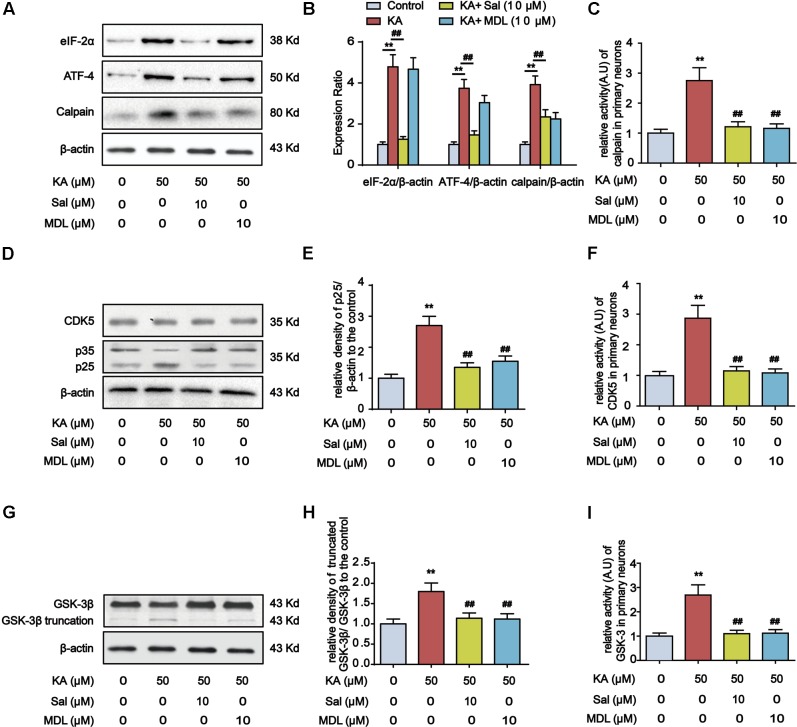
Calpain mediates ER stress-induced GSK-3β and CDK5 activation. **(A,B)** Protein levels of eIF-2α, ATF-4 and calpain in KA, Sal and/or MDL-treated primary hippocampal neurons. The primary hippocampal neurons were pre-treated with or without MDL28170 (MDL, a calpain inhibitor, 10 μM) for 1 h, and then stimulated with KA (50 μM) for 8 h. **(C)** Relative activity of calpain in KA, Sal and/or MDL-treated primary hippocampal neurons. **(D,E)** Protein levels of CDK5, p35, p25 in KA, Sal and/or MDL-treated primary hippocampal neurons. **(F)** Relative activity of CDK5 in KA, Sal and/or MDL-treated primary hippocampal neurons. **(G,H)** Protein levels of truncated-GSK-3β in KA, Sal and/or MDL-treated primary hippocampal neurons. **(I)** Relative activity of GSK-3 in KA, Sal and/or MDL-treated primary hippocampal neurons. (^∗∗^*P* < 0.01 vs. controls; ^##^*P* < 0.01 vs. the KA group; the significant difference from the respective values were determined by one-way analysis of variance test. *n* = 3 for western blotting; *N* = 5 for assay of activity).

Endoplasmic reticulum stress-dependent PERK activation leads to the elevation of the phospho-eIF2α level, which promotes the preferential synthesis of ATF-4 (Yu et al., 2016). ATF-4 functions boost the expression of calpain, triggering GSK-3β truncation (Han et al., 2014; Lu et al., 2015). Additionally, calpain can catalyze the cleavage of CDK5 neuron-specific activator p35 into more potent p25. Conversion of p35 to p25 would cause prolonged activation and mislocalization of CDK5 (Lee et al., 2000) (**Figure 9**). Accordingly, we observed that KA treatment prominently increased the expression level of eIF2α, ATF-4, and calpain in primary hippocampal neurons, whereas ER stress inhibitor Salubrinal treatment reduced the expression of them (**Figures 7A,B**).

To test the requirement of calpain in KA-induced GSK-3β and CDK5 activation, we examined the effect of calpain inhibitor MDL28170 (MDL) on the expression of p25, p35, GSK-3β and CDK5 in primary hippocampal neurons. Compared with KA-only group, Sal+KA or MDL+KA treatment decreased the expression levels of p25 and truncated GSK-3β (**Figures 7D,E,G,H**). In contrast, KA-induced increase of upstream eIF-2α or ATF-4 was not affected in calpain-inhibited neurons (MDL+KA treatment) (**Figures 7A,B**). Further analysis demonstrated that the activities of GSK-3, CDK5, and calpain were downregulated upon the treatment of either Sal or MDL (**Figures 7C,F,I**). Together our biochemical results suggested that ER stress-dependent calpain activation underlies KA-induced GSK-3β and CDK5 activation.

### Melatonin Inhibits ER Stress, Calpain Activation, and Oxidative Stress

**FIGURE 8:**
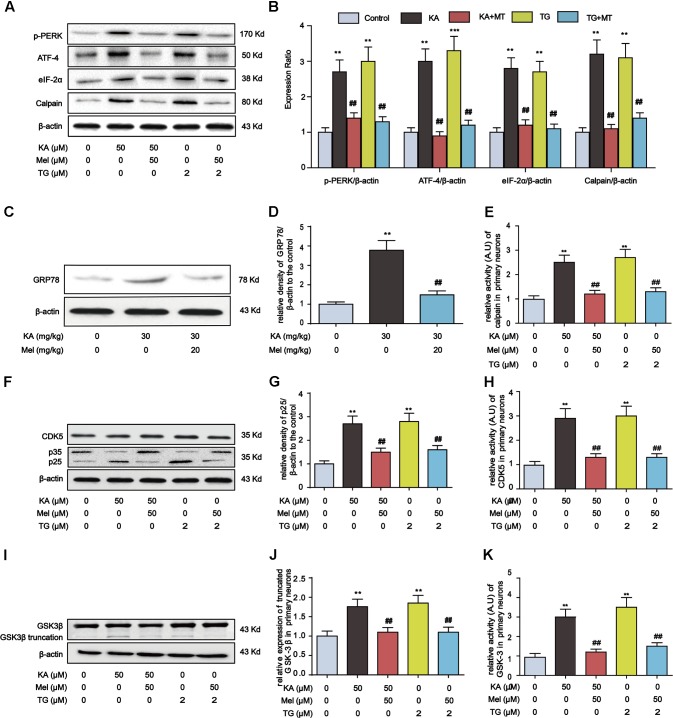
Inhibitory effects of melatonin on ER stress and GSK-3β/CDK5 activation in primary hippocampal neurons. **(A,B)** Protein levels of p-PERK, ATF-4, eIF-2α and calpain in KA, TG and/or Mel-treated primary hippocampal neurons. **(C,D)** Protein levels of GRP78 in KA and/or Mel-treated mouse hippocampus. **(E)** Relative activity of calpain in KA, TG and/or Mel-treated primary hippocampal neurons. **(F,G)** Protein levels of CDK5, p35, p25 in KA, TG and/or Mel-treated primary hippocampal neurons. **(H)** Relative activity of CDK5 in KA, TG and/or Mel-treated primary hippocampal neurons. **(I,J)** Protein levels of truncated-GSK-3β in KA, TG and/or Mel-treated primary hippocampal neurons. **(K)** Relative activity of GSK-3 in KA, TG and/or Mel-treated primary hippocampal neurons. (^∗∗^*P* < 0.01 vs. controls; ^##^*P* < 0.01 vs. the KA group; the significant difference from the respective values were determined by one-way analysis of variance test. *N* = 3 for western blotting, *n* = 5 for assay of activity).

Thus far, our evidence suggests that KA-induced ER stress triggers GSK-3β and CDK5 activation. To determine whether melatonin inhibits tau hyperphosphorylation via abolishing ER stress, we assayed the expression of proteins involved in ER stress and calpain activation after the treatment of thapsigargin (TG, an ER stress inducer). In both KA- and TG-treated groups, the expression level of p-PERK, ATF-4, eIF-2α, and calpain significantly increased. However, after the addition of melatonin, the expression of p-PERK, ATF-4, eIF-2α, and calpain reduced significantly (**Figures 8A,B**). Consistently, the increased hippocampal GRP78 level in KA-treated mice could be significantly relieved by melatonin treatment (**Figures 8C,D**).

**FIGURE 9:**
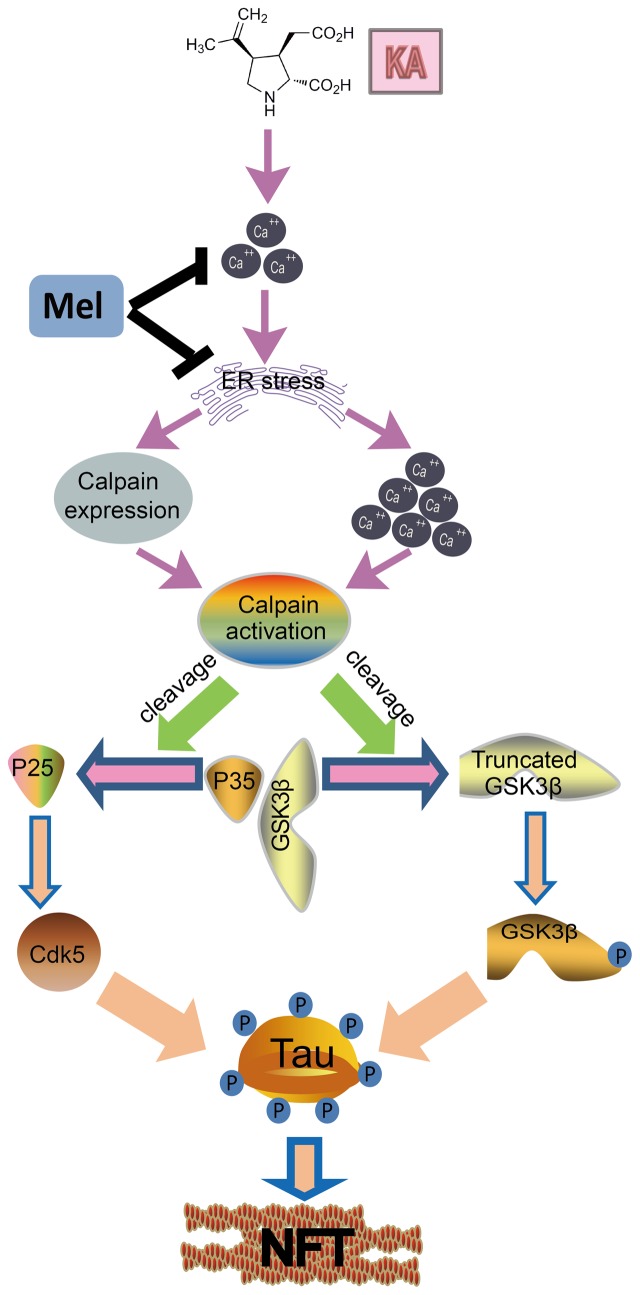
A functional model of melatonin protecting effects against KA-induced neuronal tau hyperphosphorylation and memory deficits. KA treatment triggers ER stress and causes Ca^2+^ hike, leading to calpain activity upregulation. Upregulated calpain activity eventually results in the cleavage of p35 and GSK-3β, and tau hyperphosphorylation. Melatonin inhibits KA-induced GSK-3β and CDK5 activation and tau hyperphosphorylation via alleviating ER stress. Arrows indicate positive regulation, and blocks indicate negative regulation.

It is worth noting that melatonin efficiently hampers the activity of calpain (**Figure 8E**). We then probed the expression of GSK-3β/CDK5 pathway components. While KA and the ER stress inducer TG could enhance p25 expression, the level of p25 decreased prominently upon the treatment of melatonin, suggesting the inhibitory effect of melatonin on ER stress-induced CDK5 activation (**Figures 8F–H**). Furthermore, the increased expression of truncated GSK-3β in KA or TG treated neurons was significantly reduced by melatonin treatment (**Figures 8I–K**).

Accumulating evidence showed that KA could induce oxidative stress, and melatonin treatment could effectively prevent oxidative damage (Gluck et al., 2000; Pan et al., 2015). Therefore, we tested the level of MDA, an indicator of lipid peroxidation. Also, we analyzed the activity of superoxide dismutase. We noticed that the incubation of primary hippocampal neurons with KA resulted in oxidative stress, which was characterized by a significantly increased level of MDA and a decreased activity of superoxide dismutase. However, when the cells were treated simultaneously with KA and melatonin, the up-regulated level of MDA was significantly decreased, and the activity of superoxide dismutase was considerably boosted (Supplementary Figure S1).

### Melatonin Efficiently Eases Intracellular Ca^2+^ Overload

As a glutamate analog, KA is competent to induce Ca^2+^ overloading by activating glutamate receptors (McGeer and McGeer, 1978). KA-induced extracellular Ca^2+^ influx leads to cytosolic Ca^2+^ concentration increase and ER stress (Schinder et al., 1996; Nicholls, 2004; Kim et al., 2016). Furthermore, ER dysfunction promotes Ca^2+^ release from ER and aggravate cytosolic Ca^2+^ hike (Bahar et al., 2016). To investigate the effect of melatonin on intracellular Ca^2+^ homeostasis after KA treatment, we monitored intracellular Ca^2+^ concentrations with Fura-2 AM. Supplementary Figure S3A shows the concentrations profile of Ca^2+^. KA treatment led to significant increases in cytosolic Ca^2+^ levels in both KA group and KA+Mel group concerning both amplitude and duration. However, the treatment of melatonin remarkably damper peak amplitude (5.13 ± 0.44 vs. 3.38 ± 0.25, *n* = 5, *p* < 0.05). In addition, we calculated the AUC for Ca^2+^ concentration over 8 h, and the results showed that melatonin significantly reduced the AUC by approximately 40% compared to the KA group (Supplementary Figure S3B). Therefore, melatonin could mitigate the KA-induced calpain activation by alleviating Ca^2+^ overload.

Due to the contribution of both internal and external sources of Ca^2+^, it is intriguing to determine whether ER stress-induced ER Ca^2+^ release significantly contributes to calpain activation. To this end, we used three intracellular Ca^2+^ modulators to explore the roles of [Ca^2+^]_c_ (cytosolic Ca^2+^) and [Ca^2+^]_ER_ (ER Ca^2+^ pool) in KA-treated primary hippocampal neurons. 2-APB is a specific inhibitor of inositol 1, 4, 5-trisphosphate receptor (IP3R) that promotes Ca^2+^ release from ER (Yoon et al., 2014). TG can elevate cytosolic Ca^2+^ concentration by inhibiting the ER-Ca^2+^-ATPase (Abdoul-Azize et al., 2017), while BAPTA-AM is an intracellular Ca^2+^ chelator (Liu et al., 2016). Primary hippocampal neurons were pretreated with 10 μM BAPTA-AM for 30 min, followed by the incubation with 50 μM KA for additional 8 h. Similarly, primary hippocampal neurons were co-incubated with 10 μM 2-APB or 1 μM TG and KA for 8 h. We noticed that treatment with 2-APB significantly inhibited KA-induced [Ca^2+^]_ER_ depletion (Supplementary Figure S3D) and suppressed [Ca^2+^]_c_ elevation (Supplementary Figure S3C). Instead, treatment with BAPTA-AM suppressed KA-induced [Ca^2+^]_c_ elevation while does not affect [Ca^2+^]_ER_ release (Supplementary Figures S3C,D). Moreover, treatment with TG further aggravated KA-mediated cellular Ca^2+^ overloading (Supplementary Figure S3C). These results suggested that KA can efficiently induce ER stress and Ca^2+^ release from the ER. Consistently, both the protein level and the activity of calpain were up-regulated synchronously with a KA-mediated cellular Ca^2+^ increase (**Figures 8A,E**). Therefore, although we lack direct evidence showing [Ca^2+^]_ER_ release by ER stress leads to an upregulation of calpain activity, we speculate that KA-induced ER stress plays an indispensable role in calpain activation via promoting [Ca^2+^]_c_.

## Discussion

The abnormally hyperphosphorylated tau is the major component of intracellular NFTs, which is positively correlated with the decline of memory and cognition in AD patients (Shipton et al., 2011). Multiple factors have been reported to play roles in the etiology of AD regarding initiation and progression. The excitotoxicity might occur in the early stage of the AD and contribute to the neurodegenerative process (Pallo et al., 2016). In the current study, we demonstrated that KA treatment induced ER stress and activated GSK-3β and CDK5. Moreover, we showed that KA-induced ER stress significantly activated calpain. In addition, GSK-3β or CDK5 inhibition failed to reduce ER stress, suggesting that ER stress occurs upstream of GSK-3β or CDK5. Importantly, we found that melatonin can appreciably ease KA-induced tau hyperphosphorylation and cognition disabilities via manipulating ER stress-mediated signaling pathway. In the meantime, we also noticed that the level of tau phosphorylation starts to decrease at day 7 after KA administration (**Figure 1A**). Nevertheless, ER chaperone protein GRP78 level continues to increase after day 7 (**Figure 4A**). This discrepancy could be attributed to the subsequent activation of phosphatase 2A (PP2A), leading to enhanced tau dephosphorylation. Indeed, previous studies showed that there is a dynamic fluctuation in the activity of PP2A in the KA-treated mouse brain (Liang et al., 2009).

Endoplasmic reticulum stress has been shown to contribute to various disorders in the central nervous system (Omura et al., 2013). In AD brains, p-PERK immunoreactivity was most abundant in the neurons with diffusive localization of phosphorylated tau (Hoozemans et al., 2009). Also, constant illumination resulted in ER damage, together with the decreased melatonin secretion, upregulated tau hyperphosphorylation and memory impairment (Ling et al., 2009). These studies implied the participation of ER stress in the pathophysiological process of the AD. Accordingly, our results demonstrated that the inhibition of ER stress efficiently downregulated tau hyperphosphorylation and the activation of GSK-3β and CDK5. Calpain-mediated CDK5/p35 cleavage into CDK5/p25 is necessary for tau phosphorylation (Chen et al., 2008). GSK-3β C-terminal truncation is also correlated with the over-activation of calpain in AD brains (Jin et al., 2015). ER stress-dependent activation of PERK leads to significant elevation of phospho-eIF2α, which attenuates general translation and promotes the preferential synthesis of ATF-4. ATF-4 then causes increased expression of calpain, hence triggers the proteasomal degradation of p35 and GSK-3β truncation (de la Cadena et al., 2014).

The calpain activity can be attributable to two main isoforms (calpain I and calpain II) which differed primarily in their calcium requirements. In the present study, we demonstrated that KA could simotaneouly induce cytosolic Ca^2+^ elevation and ER Ca^2+^ release. Moreover, melatonin is capable of decreasing cytosolic Ca^2+^ and ER Ca^2+^ release in KA-treated primary hippocampal neurons. Thus, we speculate that KA-induced ER stress plays an indispensable role in calpain activation via promoting cytosolic Ca^2+^ level.

It was reported that calpain I is correlated with the excitatory amino acid-induced hippocampal damage and acts as an intracellular mediator within the process (Siman et al., 1989). In the present study, we observed that p-PERK-elF2α-ATF-4 pathway mediates KA-induced upregulation of calpain, and the elevated calpain activity contributes to the activation of CDK5/GSK-3β and tau hyperphosphorylation, fortifying the mechanistic role of calpain connecting tau hyperphosphorylation with KA-induced ER stress. We also examined the role of calpain I in KA-induced tau hyperphosphorylation in N2a cells. As shown in Supplementary Figure S2, transfection of calpain I siRNA led to a significant abatement in KA-induced tau hyperphosphorylation, indicating that calpain I activation is involved in the KA-induced ER stress and tau hyperphosphorylation.

It has been speculated that tau hyperphosphorylation is a response to apoptosis. However, previous studies also showed that hippocampal neurons could survive with NFTs for over 20 years (Morsch et al., 1999). In P301S model mice, although their neurons lack cell apoptosis, there is significant tau hyperphosphorylation (Allen et al., 2002). Therefore, the correlation between tau hyperphosphorylation and apoptosis remains controversial. Unexpectedly, the pro-apoptotic factors-induced tau hyperphosphorylation was found to be accompanied by the reduced cell apoptosis (Li et al., 2007). Thus, it has been proposed that tau hyperphosphorylation may play a role in leading the neurons to abort from an acute apoptosis and triggering a chronic degenerative cell death simultaneously (Wang et al., 2014). Consistently, although prolonged ER stress could cause apoptosis, tau phosphorylation attenuated ER stress-induced apoptosis by upregulating UPR (Liu et al., 2012). Specifically, in mouse hippocampus, the activity of GSK3β/CDK5 increased 1–3 days after KA treatment. After 7 days, a reduction in GSK3β/CDK5 activation was observed, and caspase-3 activity increased. Combined with our previous findings (Xue et al., 2017), it is possible that KA-induced tau phosphorylation occurs upstream of apoptosis.

Alzheimer’s disease is the most common cause of dementia, and there is no specific and efficient cure for this disorder. Accumulating evidence suggests that melatonin plays a critical role in the development of AD and serves as a potential candidate for arresting AD-like pathological processes (Rudnitskaya et al., 2015). Melatonin can ease KA-induced tau hyperphosphorylation by inhibiting GSK-3β and CDK5 (Peng et al., 2013). Also, melatonin was reported to deactivate ER stress-mediated PERK-eIF2α-ATF-4 (Yu et al., 2016). However, it is not clear whether melatonin exerts its inhibitory effects on KA-induced tau phosphorylation and memory deficits via ER stress revising *in vivo*. Our studies showed that melatonin has beneficial effects on tau phosphorylation and memory deficits. Melatonin treatment greatly decreased the level of intracellular Ca^2+^ in KA-treated neurons. Moreover, melatonin treatment significantly reduced calpain expression/activation and ER stress in primary hippocampal neurons. Based on these results, we speculate that the cytoprotective role of melatonin could be attributed to the inhibition of ER stress and p-PERK/eIF-α/ATF-4/calpain pathway. In addition, reactive oxygen has been extensively reported to induce the deterioration of ER function and ER stress. Notably, prolonged ER stress also provoked oxidative stress, causing a toxic accumulation of ROS within the cell (Cao and Kaufman, 2014; Chaudhari et al., 2014). Thus, ER stress and oxidative stress are likely to be mutually reinforcing. Our data suggested that the antioxidative effect of melatonin could contribute to the ease of tau hyperphosphorylation. Melatonin partially reversed the KA-induced MDA elevation and the KA-suppressed superoxide dismutase activity. However, vitamin E, a recognized antioxidant, failed to affect the activities of CDK5/GSK3β and the hyperphosphorylation level of tau (data not shown), indicating that the antioxidative effect of melatonin is not sufficient to address its protective functionality.

## Author Contributions

CS and JZ carried out the cell culture and biochemical measurements, and drafted the manuscript. ZL and QC carried out the animal treatment and experiments. WH and LX carried out the GSK-3 activity assay. YW carried out the calpain activity assay. AS and JC conceived the study and participated in the coordination and preparation of the manuscript. All authors read and approved the final draft.

## Conflict of Interest Statement

The authors declare that the research was conducted in the absence of any commercial or financial relationships that could be construed as a potential conflict of interest.

## References

[B1] Abdoul-Azize S. Dubus I. Vannier J. P. (2017). Improvement of dexamethasone sensitivity by chelation of intracellular Ca^2+^ in pediatric acute lymphoblastic leukemia cells through the prosurvival kinase ERK1/2 deactivation. *Oncotarget* 8 27339–27352. 10.18632/oncotarget.16039 28423696 PMC5432339

[B2] Acuna-Castroviejo D. Escames G. Venegas C. Diaz-Casado M. E. Lima-Cabello E. Lopez L. C. (2014). Extrapineal melatonin: sources, regulation, and potential functions. *Cell. Mol. Life Sci.* 71 2997–3025. 10.1007/s00018-014-1579-2 24554058 PMC11113552

[B3] Allen B. Ingram E. Takao M. Smith M. J. Jakes R. Virdee K. (2002). Abundant tau filaments and nonapoptotic neurodegeneration in transgenic mice expressing human P301S tau protein. *J. Neurosci.* 22 9340–9351. 12417659 10.1523/JNEUROSCI.22-21-09340.2002 PMC6758022

[B4] Bahar E. Kim H. Yoon H. (2016). ER stress-mediated signaling: action potential and Ca^2+^ as key players. *Int. J. Mol. Sci.* 17:E1558. 10.3390/ijms17091558 27649160 PMC5037829

[B5] Brusco L. I. Marquez M. Cardinali D. P. (2000). Melatonin treatment stabilizes chronobiologic and cognitive symptoms in Alzheimer’s disease. *Neuro Endocrinol. Lett.* 21 39–42. 11455329

[B6] Cao S. S. Kaufman R. J. (2014). Endoplasmic reticulum stress and oxidative stress in cell fate decision and human disease. *Antioxid. Redox. Signal.* 21 396–413. 10.1089/ars.2014.5851 24702237 PMC4076992

[B7] Chaudhari N. Talwar P. Parimisetty A. Lefebvre D’hellencourt C. Ravanan P. (2014). A molecular web: endoplasmic reticulum stress, inflammation, and oxidative stress. *Front. Cell. Neurosci.* 8:213. 10.3389/fncel.2014.00213 25120434 PMC4114208

[B8] Chen X. Huang T. Zhang J. Song J. Chen L. Zhu Y. (2008). Involvement of calpain and p25 of CDK5 pathway in ginsenoside Rb1’s attenuation of beta-amyloid peptide25-35-induced tau hyperphosphorylation in cortical neurons. *Brain Res.* 1200 99–106. 10.1016/j.brainres.2007.12.029 18289510

[B9] Choi D. W. Rothman S. M. (1990). The role of glutamate neurotoxicity in hypoxic-ischemic neuronal death. *Annu. Rev. Neurosci.* 13 171–182. 10.1146/annurev.ne.13.030190.001131 1970230

[B10] Crespo-Biel N. Camins A. Canudas A. M. Pallas M. (2010). Kainate-induced toxicity in the hippocampus: potential role of lithium. *Bipolar Disord.* 12 425–436. 10.1111/j.1399-5618.2010.00825.x 20636640

[B11] de la Cadena S. G. Hernandez-Fonseca K. Camacho-Arroyo I. Massieu L. (2014). Glucose deprivation induces reticulum stress by the PERK pathway and caspase-7- and calpain-mediated caspase-12 activation. *Apoptosis* 19 414–427. 10.1007/s10495-013-0930-7 24185830

[B12] Gluck M. R. Jayatilleke E. Shaw S. Rowan A. J. Haroutunian V. (2000). CNS oxidative stress associated with the kainic acid rodent model of experimental epilepsy. *Epilepsy Res.* 39 63–71. 10.1016/S0920-1211(99)00111-4 10690755

[B13] Grundke-Iqbal I. Iqbal K. Quinlan M. Tung Y. C. Zaidi M. S. Wisniewski H. M. (1986). Microtubule-associated protein tau. A component of Alzheimer paired helical filaments. *J. Biol. Chem.* 261 6084–6089. 3084478

[B14] Hadj Ayed Tka K. Mahfoudh Boussaid A. Zaouali M. A. Kammoun R. Bejaoui M. Ghoul Mazgar S. (2015). Melatonin modulates endoplasmic reticulum stress and Akt/GSK3-beta signaling pathway in a rat model of renal warm ischemia reperfusion. *Anal. Cell. Pathol.* 2015:635172. 10.1155/2015/635172 26229743 PMC4502281

[B15] Han G. Casson R. J. Chidlow G. Wood J. P. (2014). The mitochondrial complex I inhibitor rotenone induces endoplasmic reticulum stress and activation of GSK-3beta in cultured rat retinal cells. *Invest. Ophthalmol. Vis. Sci.* 55 5616–5628. 10.1167/iovs.14-14371 25082888

[B16] Hernandez F. Avila J. (2008). Tau aggregates and tau pathology. *J. Alzheimers Dis.* 14 449–452. 10.3233/JAD-2008-14414 18688097

[B17] Hoozemans J. J. M. Van Haastert E. S. Nijholt D. A. T. Rozemuller A. J. M. Eikelenboom P. Scheper W. (2009). The unfolded protein response is activated in pretangle neurons in Alzheimer’s disease hippocampus. *Am. J. Pathol.* 174 1241–1251. 10.2353/ajpath.2009.080814 19264902 PMC2671357

[B18] Hoozemans J. J. M. Veerhuis R. Van Haastert E. S. Rozemuller J. M. Baas F. Eikelenboom P. (2005). The unfolded protein response is activated in Alzheimer’s disease. *Acta Neuropathol.* 110 165–172. 10.1007/s00401-005-1038-0 15973543

[B19] Isaev N. K. Stelmashook E. V. Dirnagl U. Andreeva N. A. Manuhova L. Vorobjev V. S. (2002). Neuroprotective effects of the antifungal drug clotrimazole. *Neuroscience* 113 47–53. 10.1016/S0306-4522(02)00164-1 12123683

[B20] Jain A. Sharma D. Suhalka P. Sukhwal P. Bhatnagar M. (2013). Changes in the density of nitrergic neurons in the hippocampus of rats following kainic acid and melatonin administration. *Physiol. Res.* 62 197–203. 23234414 10.33549/physiolres.932295

[B21] Jin N. Yin X. Yu D. Cao M. Gong C. X. Iqbal K. (2015). Truncation and activation of GSK-3beta by calpain I: a molecular mechanism links to tau hyperphosphorylation in Alzheimer’s disease. *Sci. Rep.* 5:8187. 10.1038/srep08187 25641096 PMC4313118

[B22] Kim H. Lee J. Y. Park K. J. Kim W. H. Roh G. S. (2016). A mitochondrial division inhibitor, Mdivi-1, inhibits mitochondrial fragmentation and attenuates kainic acid-induced hippocampal cell death. *BMC Neurosci.* 17:33. 10.1186/s12868-016-0270-y 27287829 PMC4902937

[B23] Kim J. S. Heo R. W. Kim H. Yi C. O. Shin H. J. Han J. W. (2014). Salubrinal, ER stress inhibitor, attenuates kainic acid-induced hippocampal cell death. *J. Neural. Transm.* 121 1233–1243. 10.1007/s00702-014-1208-0 24728926

[B24] Kobe H. Nakai A. Koshino T. Araki T. (2002). Effect of regular maternal exercise on lipid peroxidation levels and antioxidant enzymatic activities before and after delivery. *J. Nippon Med. Sch.* 69 542–548. 10.1272/jnms.69.542 12646986

[B25] Lee M. S. Kwon Y. T. Li M. Peng J. Friedlander R. M. Tsai L. H. (2000). Neurotoxicity induces cleavage of p35 to p25 by calpain. *Nature* 405 360–364. 10.1038/35012636 10830966

[B26] Li H. L. Wang H. H. Liu S. J. Deng Y. Q. Zhang Y. J. Tian Q. (2007). Phosphorylation of tau antagonizes apoptosis by stabilizing beta-catenin, a mechanism involved in Alzheimer’s neurodegeneration. *Proc. Natl. Acad. Sci. U.S.A.* 104 3591–3596. 10.1073/pnas.0609303104 17360687 PMC1805527

[B27] Liang Z. Liu F. Iqbal K. Grundke-Iqbal I. Gong C. X. (2009). Dysregulation of tau phosphorylation in mouse brain during excitotoxic damage. *J. Alzheimers Dis.* 17 531–539. 10.3233/JAD-2009-1069 19363259 PMC2829309

[B28] Ling Z. Q. Tian Q. Wang L. Fu Z. Q. Wang X. C. Wang Q. (2009). Constant illumination induces Alzheimer-like damages with endoplasmic reticulum involvement and the protection of melatonin. *J. Alzheimers Dis.* 16 287–300. 10.3233/JAD-2009-0949 19221418

[B29] Liu C. Ye Y. Zhou Q. Zhang R. Zhang H. Liu W. (2016). Crosstalk between Ca^2+^ signaling and mitochondrial H_2_O_2_ is required for rotenone inhibition of mTOR signaling pathway leading to neuronal apoptosis. *Oncotarget* 7 7534–7549. 10.18632/oncotarget.7183 26859572 PMC4884936

[B30] Liu R. Y. Zhou J. N. Van Heerikhuize J. Hofman M. A. Swaab D. F. (1999). Decreased melatonin levels in postmortem cerebrospinal fluid in relation to aging, Alzheimer’s disease, and apolipoprotein E-epsilon4/4 genotype. *J. Clin. Endocrinol. Metab.* 84 323–327. 9920102 10.1210/jcem.84.1.5394

[B31] Liu X. A. Song J. Jiang Q. Wang Q. Tian Q. Wang J. Z. (2012). Expression of the hyperphosphorylated tau attenuates ER stress-induced apoptosis with upregulation of unfolded protein response. *Apoptosis* 17 1039–1049. 10.1007/s10495-012-0744-z 22802092

[B32] Lu T. H. Su C. C. Tang F. C. Chen C. H. Yen C. C. Fang K. M. (2015). Chloroacetic acid triggers apoptosis in neuronal cells via a reactive oxygen species-induced endoplasmic reticulum stress signaling pathway. *Chem. Biol. Interact.* 225 1–12. 10.1016/j.cbi.2014.10.022 25451595

[B33] Marklund S. Marklund G. (1974). Involvement of the superoxide anion radical in the autoxidation of pyrogallol and a convenient assay for superoxide dismutase. *Eur. J. Biochem.* 47 469–474. 10.1111/j.1432-1033.1974.tb03714.x 4215654

[B34] McGeer E. G. McGeer P. L. (1978). Some factors influencing the neurotoxicity of intrastriatal injections of kainic acid. *Neurochem. Res.* 3 501–517. 10.1007/BF00966331 34114

[B35] Medeiros R. Baglietto-Vargas D. Laferla F. M. (2011). The role of tau in Alzheimer’s disease and related disorders. *CNS Neurosci. Ther.* 17 514–524. 10.1111/j.1755-5949.2010.00177.x 20553310 PMC4072215

[B36] Milatovic D. Zivin M. Gupta R. C. Dettbarn W. D. (2001). Alterations in cytochrome c oxidase activity and energy metabolites in response to kainic acid-induced status epilepticus. *Brain Res.* 912 67–78. 10.1016/S0006-8993(01)02657-9 11520494

[B37] Morris M. Maeda S. Vossel K. Mucke L. (2011). The many faces of tau. *Neuron* 70 410–426. 10.1016/j.neuron.2011.04.009 21555069 PMC3319390

[B38] Morsch R. Simon W. Coleman P. D. (1999). Neurons may live for decades with neurofibrillary tangles. *J. Neuropathol. Exp. Neurol.* 58 188–197. 10.1097/00005072-199902000-00008 10029101

[B39] Nicholls D. G. (2004). Mitochondrial dysfunction and glutamate excitotoxicity studied in primary neuronal cultures. *Curr. Mol. Med.* 4 149–177. 10.2174/1566524043479239 15032711

[B40] Omura T. Kaneko M. Okuma Y. Matsubara K. Nomura Y. (2013). Endoplasmic reticulum stress and Parkinson’s disease: the role of HRD1 in averting apoptosis in neurodegenerative disease. *Oxid. Med. Cell. Longev.* 2013:239854. 10.1155/2013/239854 23710284 PMC3654363

[B41] Pallo S. P. Dimaio J. Cook A. Nilsson B. Johnson G. V. (2016). Mechanisms of tau and Abeta-induced excitotoxicity. *Brain Res.* 1634 119–131. 10.1016/j.brainres.2015.12.048 26731336 PMC4779680

[B42] Pan X. Zhu L. Lu H. Wang D. Lu Q. Yan H. (2015). Melatonin attenuates oxidative damage induced by acrylamide in vitro and in vivo. *Oxid. Med. Cell. Longev.* 2015:703709. 10.1155/2015/703709 26185593 PMC4491391

[B43] Peng C. X. Hu J. Liu D. Hong X. P. Wu Y. Y. Zhu L. Q. (2013). Disease-modified glycogen synthase kinase-3beta intervention by melatonin arrests the pathology and memory deficits in an Alzheimer’s animal model. *Neurobiol. Aging* 34 1555–1563. 10.1016/j.neurobiolaging.2012.12.010 23402899

[B44] Pigino G. Paglini G. Ulloa L. Avila J. Caceres A. (1997). Analysis of the expression, distribution and function of cyclin dependent kinase 5 (cdk5) in developing cerebellar macroneurons. *J. Cell Sci.* 110(Pt 2), 257–270. 9044056 10.1242/jcs.110.2.257

[B45] Rudnitskaya E. A. Muraleva N. A. Maksimova K. Y. Kiseleva E. Kolosova N. G. Stefanova N. A. (2015). Melatonin attenuates memory impairment, amyloid-beta accumulation, and neurodegeneration in a rat model of sporadic Alzheimer’s disease. *J. Alzheimers Dis.* 47 103–116. 10.3233/JAD-150161 26402759

[B46] Salminen A. Kauppinen A. Suuronen T. Kaarniranta K. Ojala J. (2009). ER stress in Alzheimer’s disease: a novel neuronal trigger for inflammation and Alzheimer’s pathology. *J. Neuroinflammation* 6:41. 10.1186/1742-2094-6-41 20035627 PMC2806266

[B47] Schinder A. F. Olson E. C. Spitzer N. C. Montal M. (1996). Mitochondrial dysfunction is a primary event in glutamate neurotoxicity. *J. Neurosci.* 16 6125–6133. 8815895 10.1523/JNEUROSCI.16-19-06125.1996 PMC6579180

[B48] Sharma S. Sarkar J. Haldar C. Sinha S. (2014). Melatonin reverses Fas, E2F-1 and endoplasmic reticulum stress mediated apoptosis and dysregulation of autophagy induced by the herbicide atrazine in murine splenocytes. *PLOS ONE* 9:e108602. 10.1371/journal.pone.0108602 25259610 PMC4178181

[B49] Shipton O. A. Leitz J. R. Dworzak J. Acton C. E. J. Tunbridge E. M. Denk F. (2011). Tau protein is required for amyloid β-induced impairment of hippocampal long-term potentiation. *J. Neurosci.* 31 1688–1692. 10.1523/JNEUROSCI.2610-10.2011 21289177 PMC3836238

[B50] Siman R. Noszek J. C. Kegerise C. (1989). Calpain I activation is specifically related to excitatory amino acid induction of hippocampal damage. *J. Neurosci.* 9 1579–1590. 2542478 10.1523/JNEUROSCI.09-05-01579.1989 PMC6569848

[B51] Tripathi P. P. Santorufo G. Brilli E. Borrelli E. Bozzi Y. (2010). Kainic acid-induced seizures activate GSK-3beta in the hippocampus of D2R-/- mice. *Neuroreport* 21 846–850. 10.1097/WNR.0b013e32833d5891 20625330

[B52] Wang H. Wang Z. K. Jiao P. Zhou X. P. Yang D. B. Wang Z. Y. (2015). Redistribution of subcellular calcium and its effect on apoptosis in primary cultures of rat proximal tubular cells exposed to lead. *Toxicology* 333 137–146. 10.1016/j.tox.2015.04.015 25921245

[B53] Wang J. Z. Wang Z. H. Tian Q. (2014). Tau hyperphosphorylation induces apoptotic escape and triggers neurodegeneration in Alzheimer’s disease. *Neurosci. Bull.* 30 359–366. 10.1007/s12264-013-1415-y 24627329 PMC5562660

[B54] Wu Y. H. Swaab D. F. (2005). The human pineal gland and melatonin in aging and Alzheimer’s disease. *J. Pineal Res.* 38 145–152. 10.1111/j.1600-079X.2004.00196.x 15725334

[B55] Xue F. Shi C. Chen Q. Hang W. Xia L. Wu Y. (2017). Melatonin mediates protective effects against kainic acid-induced neuronal death through safeguarding ER stress and mitochondrial disturbance. *Front. Mol. Neurosci.* 10:49. 10.3389/fnmol.2017.00049 28293167 PMC5329003

[B56] Yoon M. J. Lee A. R. Jeong S. A. Kim Y. S. Kim J. Y. Kwon Y. J. (2014). Release of Ca^2+^ from the endoplasmic reticulum and its subsequent influx into mitochondria trigger celastrol-induced paraptosis in cancer cells. *Oncotarget* 5 6816–6831. 10.18632/oncotarget.2256 25149175 PMC4196165

[B57] Yu L. Li B. Zhang M. Jin Z. Duan W. Zhao G. (2016). Melatonin reduces PERK-eIF2alpha-ATF4-mediated endoplasmic reticulum stress during myocardial ischemia-reperfusion injury: role of RISK and SAFE pathways interaction. *Apoptosis* 21 809–824. 10.1007/s10495-016-1246-1 27170343

[B58] Zhang X. M. Zhu J. (2011). Kainic acid-induced neurotoxicity: targeting glial responses and glia-derived cytokines. *Curr. Neuropharmacol.* 9 388–398. 10.2174/157015911795596540 22131947 PMC3131729

[B59] Zhou J. N. Liu R. Y. Kamphorst W. Hofman M. A. Swaab D. F. (2003). Early neuropathological Alzheimer’s changes in aged individuals are accompanied by decreased cerebrospinal fluid melatonin levels. *J. Pineal Res.* 35 125–130. 10.1034/j.1600-079X.2003.00065.x 12887656

