# Supplementary material for: Melatonin Mitigates Kainic Acid-Induced Neuronal Tau Hyperphosphorylation and Memory Deficits through Alleviating ER Stress
Source: Front Mol Neurosci. 2018 Jan 24;11:5. doi: 10.3389/fnmol.2018.00005 (PMC5787934; doi:10.3389/fnmol.2018.00005)
Supplement: Supplementary file 1 [file Data_Sheet_1.PDF]

Melatonin mitigates kainic acid-induced neuronal tau hyperphosphorylation and memory deficits through alleviating ER stress

Cai Shi[a#], Jia Zeng[a#], Zixi Li[d], Qingjie Chen[a], Weijian Hang[a], Liangtao Xia[a], Yue Wu[a], Juan Chen[a,b]*, Anbing Shi[a,b,c]*

[a] Department of Biochemistry and Molecular Biology, School of Basic Medicine and the Collaborative Innovation Center for Brain Science, Tongji Medical College, Huazhong University of Science and Technology, Wuhan, 430030, Hubei, China

[b] Institute for Brain Research, Huazhong University of Science and Technology, Wuhan, 430030, Hubei, China

[c] Key Laboratory of Neurological Disease of National Education Ministry, Tongji Medical College, Huazhong University of Science and Technology, Wuhan, 430030, Hubei, China

[d] Department of Clinical laboratory, Wuhan No. 1 Hospital, Tongji Medical College, Huazhong University of Science and Technology, Wuhan, 430030, Hubei, China

[e] Department of Histology and Embryology, School of Basic Medicine, Tongji Medical College, Huazhong University of Science and Technology, Wuhan 430030, Hubei, China

Supplemental Figures

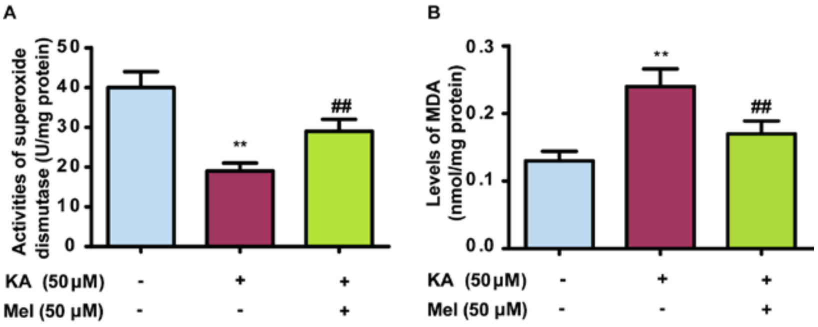

Figure S1. Protective effects of melatonin on KA-induced oxidative damage in primary neurons.

(A) Activities of Superoxide dismutase in primary neurons. (B) Levels of MDA in primary neurons. (**P<0.01 vs. controls; ##P<0.01 vs. the KA group; n=5)

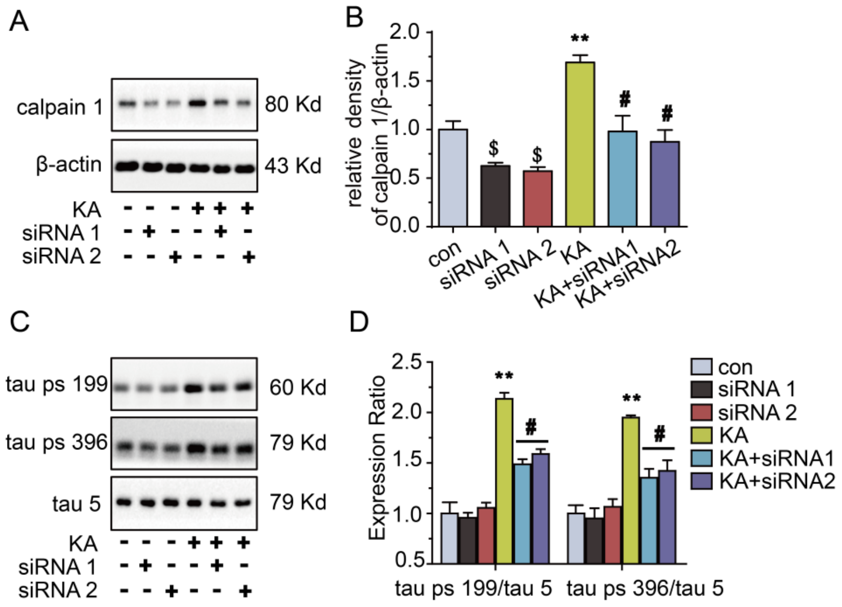

Figure S2: Transfection of calpain I siRNA inhibited KA-induced tau hyperphosphorylation.

N2a were transfected with control- or calpain I-siRNA for 36 hours and then stimulated with KA (50 μM) for 8 hours. The proteins (A-B. calpain I; C-D. p-tau199 and p-tau396) were determined by Western blotting. (Data are shown as the mean ± SEM (n=3). *P<0.05, $^{\$}$P<0.05 and **P<0.01 as compared with control group;

#P<0.05 compared with the KA-treated group).

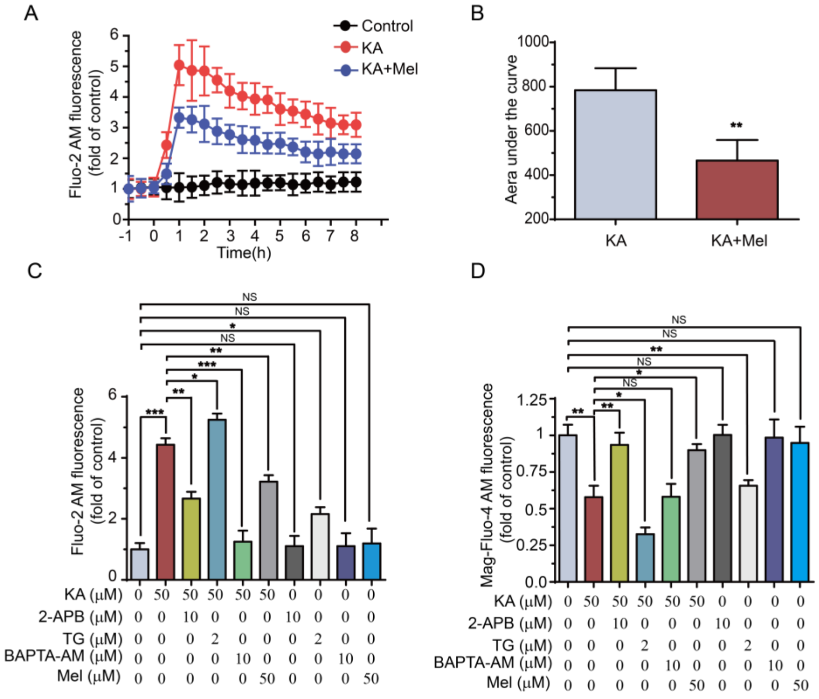

Figure. S3. Melatonin inhibits intracellular Ca^{2+} overload after KA treatment. (A) Primary hippocampal neurons were pre-treated with or without melatonin (50 μM) for 1 hour and then stimulated with KA (50μM) for 8 hours .The intracellular Ca^{2+} concentration ($[Ca^{2+}]_c$) was determined by Fura-2 AM up to 8 hours. (B) Total area under the curve of each phase was calculated. (C) Primary hippocampal neurons were treated with 50 μM KA for 8 hours in the presence or absence of 10 μM 2-APB (co-incubation for the entire course), 1 μM TG (co-incubation for the entire course), 10 μM BAPTA-AM (30 min pre-incubation before KA treatment) and 50 μM melatonin (1 hour pre-incubation before KA treatment) to assess the changes of $[Ca^{2+}]_c$ by Fura-2 AM. (D) The changes of $[Ca^{2+}]_{ER}$ assessed by Mag-Fluo-4. Values of fluorescence intensity of Fluo-2 AM are quantified in a relative way to its respective control, whose value is set at one. Values of fluorescence intensity of Mag-Fluo-4 are quantified in a relative way to its respective control. (Data are shown as the mean ± SEM (n = 6). ns not significant, *P < 0.05, **P < 0.01).
